# Occurrence and relative risks for non-vertebral fractures in patients with ankylosing spondylitis compared with the general population: a register-based study from Sweden

**DOI:** 10.1136/rmdopen-2022-002753

**Published:** 2023-02-14

**Authors:** Karin Bengtsson, Johan Askling, Mattias Lorentzon, Björn Rosengren, Anna Deminger, Eva Klingberg, Lennart Jacobsson, Helena Forsblad-d'Elia

**Affiliations:** 1 Department of Rheumatology and Inflammation Research, Institute of Medicine, Sahlgrenska Academy at University of Gothenburg, Göteborg, Sweden; 2 Department of Rheumatology, Sahlgrenska University Hospital, Region Västra Götaland, Göteborg, Sweden; 3 Clinical Epidemiology Division, Department of Medicine Solna, Karolinska Institutet, Stockholm, Sweden; 4 Sahlgrenska Osteoporosis Center, Department of Internal Medicine and Clinical Nutrition, Institute of Medicine, Sahlgrenska Academy at University of Gothenburg, Göteborg, Sweden; 5 Department of Geriatric Medicine, Sahlgrenska University Hospital, Region Västra Götaland, Mölndal, Sweden; 6 Clinical and Molecular Osteoporosis Research Unit, Departments of Orthopedics and Clinical Sciences, Lund University, Malmö, Sweden; 7 Department of Orthopedics, Skåne University Hospital, Malmö, Sweden

**Keywords:** spondylitis, ankylosing, epidemiology, osteoporosis

## Abstract

**Objectives:**

To estimate the incidence of non-vertebral fractures in ankylosing spondylitis (AS) compared with the general population.

**Methods:**

Nationwide register-based cohort study including patients with AS (n=11 611, 65% men, mean age 48 years), and matched general population controls (n=58 050). Five prespecified fracture outcomes: (1) non-vertebral; (2) fracture of the proximal humerus, distal forearm or hip; (3) proximal humerus; (4) distal forearm and (5) hip) were identified through register linkages with follow-up 2007–2016. We used Poisson regression to calculate incidence rates (IRs), number of fractures per 1000 person-years at risk and IR ratios (IRRs), overall and by sex and age. IRRs were adjusted for history of any prior fracture.

**Results:**

IRs (men/women) for non-vertebral fracture in AS were 11.9 (95% CI 11.0 to 12.9)/14.5 (95% CI 13.1 to 16.1) and in controls 10.0 (95% CI 9.7 to 10.4)/11.8 (95% CI 11.1 to 12.4), IRR (men/women) 1.2 (95% CI 1.1 to 1.3)/1.2 (95% CI 1.1 to 1.4). IRs (men/women) for fractures of the humerus, forearm or hip in AS were 4.0 (95% CI 3.5 to 4.6)/6.3 (95% CI 5.4 to 7.3) and in controls 2.7 (95% CI 2.5 to 2.9)/5.5 (95% CI 5.1 to 6.0), IRR (men/women) 1.5 (95% CI 1.3 to 1.7)/1.1 (95% CI 0.9 to 1.3). IRRs were statistically significantly elevated in men with AS versus controls for forearm fracture (1.4 (95% CI 1.1 to 1.7)) and hip fracture (1.8 (95% CI 1.4 to 2.3)), whereas not in women with AS where the IRRs were 1.1 (95% CI 0.9 to 1.4) and 1.0 (95% CI 0.6 to 1.4). For humerus fracture, IRRs were 1.4 (95% CI 0.99 to 1.9) in men with AS versus controls and 1.1 (95% CI 0.8 to 1.6) in women.

**Conclusions:**

Both men and women with AS have a slightly higher risk of non-vertebral fractures than the general population. A statistically significantly higher risk of fractures of the proximal humerus, distal forearm or hip was found in men with AS in comparison to general population, where the relative risk was especially pronounced for hip fracture.

**WHAT IS ALREADY KNOWN ON THIS TOPIC**

- Vertebral fractures are known complications of ankylosing spondylitis (AS), whereas the risk of other fractures is less studied.

**WHAT THIS STUDY ADDS**

- In this nationwide, register-based cohort study of patients with AS and controls from the general population we investigated the risks of non-vertebral fractures with special focus on fractures of the proximal humerus, distal forearm and hip.
- We found a slightly increased risk of non-vertebral fractures in both men and women with AS compared with general population. Regarding fractures of the humerus, forearm or hip, a statistically significantly increased risk was found in men with AS in comparison to general population and the relative risk was especially pronounced for hip fracture.

**HOW THIS STUDY MIGHT AFFECT RESEARCH, PRACTICE OR POLICY**

- The results of this study acknowledge the importance of fracture risk assessment in patients with AS.

## Introduction

Characteristic signs of ankylosing spondylitis (AS) include excessive new bone formation as well as erosions, resulting in a rigid spine with loss of mobility and in some patients a completely ankylosed spine.^1^ Osteoporosis is frequently observed in AS and, according to a meta-analysis with prevalence ranging between 11.7% and 34.4%.^2^ Register-based observational studies have reported a higher risk of diagnosed osteoporosis in AS compared with the general population.^3 4^ Further, significantly lower bone mineral density (BMD) has been observed in patients with AS compared with healthy controls.^5–7^ Importantly, decreased BMD in lumbar spine and femoral neck were found in around half of all patients within 10 years after the AS diagnosis.^8^ Vertebral fractures are also common and prevalent in 11%–20% of patients included in AS cohorts.^9–14^ In comparison with the general population, an increased risk of vertebral fractures has consistently been shown in previous studies.^13 15–21^ On the contrary, studies investigating risk of non-vertebral fractures in AS are fewer, and results inconsistent.^15–19 21^ In addition, only one of the previous studies has investigated the risk of non-vertebral fractures separately in women and men.^21^

We; therefore, performed a register-based national study to investigate the risks of non-vertebral fractures with special focus on fracture sites commonly associated with osteoporosis (proximal humerus, distal forearm and hip) in patients with AS, overall and stratified by sex and age groups, and to compare with the risks in the general population.

## Methods

### Study design

Nationwide matched register-based cohort study.

### Register sources

The National Patient Register (NPR) includes data on Swedish inpatient care with nationwide coverage from 1987 and specialised outpatient care from 2001 and forward. For each physician visit and hospital discharge, the primary and secondary diagnoses are registered according to the International Classification of Diseases (ICD) coding system as well as surgical procedure codes. NPR was used to identify patients, baseline comorbidities and the different fracture outcomes. The Swedish Population Register contains demographic data for all Swedish residents and was used to identify the matched controls and reasons for censoring (death and emigration) during follow-up. The Prescribed Drug Register (PDR) encompasses information on all dispensed prescription from July 2005 and thereafter. Medical treatment related to AS and osteoporosis were identified from PDR according to Anatomical Therapeutic Chemical Classification (ATC) codes. Medications administered at healthcare units are not identified in PDR. Intravenously administered TNF inhibitors were therefore captured from the Swedish Rheumatology Quality Register (SRQ). SRQ has a high coverage (86%) of patients with spondyloarthritis (SpA), including AS, treated with TNF inhibitors.^22^ The linkage of the registers was possible through the personal identification number, a unique number issued to all Swedish residents.

### Study population

All patients aged ≥18 years with a diagnostic code for AS (ICD version 10 (ICD-10): M45.9) reported from at least one physician visit in rheumatology or internal medicine outpatient care 2001–2015, alive and living in Sweden at start of follow-up, were identified and included in the AS cohort. To further strengthen the validity of the AS case definition, patients with a diagnostic code 2001 through 2015 for rheumatoid arthritis, psoriatic arthritis or systemic lupus erythematosus after the AS diagnosis were excluded.

For each index patient, up to five controls matched for age, sex and region of residency were identified from the Swedish Population Register. At start of follow-up, the controls had to be alive, live in Sweden and not fulfil the case definition. The controls were allowed to have inflammatory conditions other than AS.

The follow-up started on 1 January 2007 or 3 months after the first reported diagnosis of AS if this occurred later. Controls started the follow-up at the same time as their index patient. This strategy guaranteed at least 6 years of prefollow-up data from the specialised outpatient part of NPR (to identify comorbid conditions including prior fractures at start of follow-up) and at least 1 year of prefollow-up data from the PDR (to identify medication use at start of follow-up) for all included patients and controls. The 3-month lag period was applied to rule out immediate detection or reporting biases between the AS diagnosis and the fracture outcomes.

Patients and controls were followed in the registers until the first occurrence of the fracture outcome of interest or censoring due to loss of follow-up. Reasons for censoring were death, emigration or end of study, which was set to 31 December 2016. Specifically for the controls, the follow-up also ended if they were diagnosed with AS.

### Non-vertebral fracture outcomes

All available physician visits in specialised outpatient care and hospital discharges 2001–2016 with reported non-vertebral fractures were identified in the NPR according to specified ICD-10 codes (online supplemental table 1) and further subdivided in site of fracture based on a two-digit system (S22, S32, S42, S52, S62, S72, S82 and S92). The fractures were identified irrespective of cause of injury. Neither fractures of the skull, fingers and toes, nor unspecified ICD codes with poor discrimination between vertebral and non-vertebral fractures were included in the non-vertebral fracture outcome. In order to minimise the risk of including readmissions of fractures occurring before start of follow-up, a lag period of at least 5 months was required between fractures at the same site (according to the two-digit system) to be counted as a new incident fracture during follow-up. This lag period was chosen based on a previous register-based study, which used medical records and X-ray reports to define the optimal time point to identify true incident fractures.^23 24^ Also, all subsequent fracture of the same site (according to the two-digit system) with an accompanying code for ‘control after fracture’ (ICD version 10: Z094) were excluded irrespective of time between the registered fractures. We thereafter identified the first non-vertebral fracture occurring during follow-up for each individual. Correspondingly, the first fracture of the proximal humerus, distal forearm and hip, respectively, occurring during follow-up were identified according to specified ICD codes and for the hip fracture outcome, specified surgery codes were required as complement. These fractures were categorised both as a composite outcome, fractures of the humerus, forearm or hip, and as three separate outcomes (fracture of the humerus, fracture of the forearm and fracture of the hip, respectively). Only the first fracture per fracture outcome, occurring during follow-up, was counted. As secondary fracture outcomes, we also identified the first fracture for each site based on the two-digit system of the ICD-10 (S22, S32, S42, S52, S62, S72, S82 and S92) during follow-up.

10.1136/rmdopen-2022-002753.supp1 Supplementary data

### Baseline characteristics

To characterise the cohorts at start of follow-up, comorbid conditions and medications of relevance for AS, and history of fractures, were extracted from NPR and PDR according to specified ICD and ATC codes (online supplemental table 1). The comorbidities and medications had to be recorded in the registers within the preceding six (comorbidities) and one (medication use) year, respectively, to be counted as present at start of follow-up. Here, we also retrieved data for prior vertebral fractures within the preceding 6 years and any prior fracture, which in addition to the definition of non-vertebral fractures (described in the previous section) also included ICD codes corresponding to a vertebral or unspecified fracture.

### Statistics

Categorical data are reported as frequencies (percent) and continuous data as means (SDs). The five different primary fracture outcomes as well as the secondary fracture outcomes were examined in separate analyses. For each fracture outcome, time to the first outcome of interest or censoring was calculated. Consequently, each individual could contribute with maximum one event per fracture outcome. The incidence rates (IRs), overall and stratified by sex and age groups (18–29 years, 30–39 years, 40–49 years, 50–59 years, 60–69 years, 70–79 years, 80 years and above), were calculated as the number of registered events (per outcome) divided by the corresponding follow-up time (per outcome), and presented as number of fractures per 1000 person-years at risk in AS and matched controls, respectively. 95% CIs were calculated assuming a Poisson distribution of the observed events. Kaplan-Meier curves were plotted for each fracture outcome. For the comparison between AS and matched controls (reference), IR ratios (IRRs) with 95% CI were assessed through Poisson regression analyses, overall and stratified by sex and age groups. If the number of fracture outcomes was less than ten per strata, comparative analyses were not performed. Since prior fracture is a strong risk factor for a subsequent fracture, the regression analyses were adjusted for any prior fracture at start of follow-up.^25^ The clustering between index patient and matched controls were kept in all comparative analyses. The overall and sex-stratified models were checked for correlation and interaction between AS status and sex, age and any prior fracture.

### Sensitivity analyses

As a sensitivity analysis, the IRs and IRRs were also determined for the subgroup of patients and their matched controls without a history of any fracture within the six preceding years before start of follow-up. In a second sensitivity analysis, we required 12 months between fractures at the same site (according to the two-digit system) to be counted as a new incident fracture during follow-up and recalculated the IRs and crude IRRs for non-vertebral fracture, humerus fracture, forearm fracture and hip fracture in AS and controls. Lastly, we also identified the first vertebral fracture according to specified ICD codes (online supplemental table 1) during follow-up and calculated IRs and IRRs, adjusted for any prior fracture.

SAS V.9.4 (SAS Institute) was used to handle the data and statistical analyses.

## Results

We included 11 611 patients with AS (65.5% men, mean age 48 (SD 15) years) and 58 050 age-matched, sex-matched and geography-matched controls. The sex-stratified characteristics of the patients and controls at start of follow-up are described in table 1. Overall, 807 (7.0%) of the AS patients and 3 201 (5.5%) of the controls had experienced any prior fracture within the preceding 6 years and the corresponding data for vertebral fractures were 184 (1.6%) and 244 (0.4%), respectively. Furthermore, 354 (3.0%) patients and 354 (0.6%) controls were prescribed an active medical treatment against osteoporosis, almost exclusively a bisphosphonate. Nearly half of the patient cohort (n=5498, 47%), together with their matched controls, started the follow-up in 2007 and constituted of prevalent AS identified 2001–2006. The mean follow-up time in both AS and their matched controls were 6.9 (3.2) person-years at risk. During follow-up, 814 (7.0%) of the patients died and 131 (1.1%) emigrated. Among the controls, 3209 (5.5%) died, 887 (1.5%) emigrated and 53 (0.1%) were diagnosed with AS after study entry (and censored at that time point).

**Table 1:** Characteristics of the patients with AS and matched control at start of follow-up

	AS		Matched controls	
	Men N=7605	Women N=4006	Men N=38 020	Women N=20 030
Age, mean (SD)	49 (15)	47 (14)	49 (15)	47 (14)
18-29 years, n (%)	867 (11.4)	503 (12.6)	4330 (11.4)	2515 (12.6)
30–39 years, n (%)	1429 (18.8)	806 (20.1)	7145 (18.8)	4030 (20.1)
40–49 years, n (%)	1621 (21.3)	992 (24.8)	8105 (21.3)	4960 (24.8)
50–59 years, n (%)	1654 (21.7)	850 (21.2)	8270 (21.8)	4250 (21.2)
60–69 years, n (%)	1410 (18.5)	593 (14.8)	7050 (18.5)	2965 (14.8)
70–79 years, n (%)	524 (6.9)	203 (5.1)	2620 (6.9)	1015 (5.1)
80+ years, n (%)	100 (1.3)	59 (1.5)	500 (1.3)	295 (1.5)
Prior medical conditions				
Any fracture	538 (7.1)	269 (6.7)	2153 (5.7)	1048 (5.2)
Vertebral fracture	137 (1.8)	47 (1.2)	170 (0.4)	74 (0.4)
Non-vertebral fracture	422 (5.5)	234 (5.8)	2002 (5.3)	988 (4.9)
Fracture of the humerus, forearm or hip	112 (1.5)	105 (2.6)	429 (1.1)	417 (2.1)
Proximal humerus fracture	25 (0.3)	32 (0.8)	80 (0.2)	82 (0.4)
Distal forearm fracture	66 (0.9)	63 (1.6)	274 (0.7)	296 (1.5)
Hip fracture	23 (0.3)	14 (0.3)	84 (0.2)	60 (0.3)
Fall injury	848 (11.2)	439 (11.0)	3744 (9.8)	1753 (8.8)
Osteoporosis	75 (1.0)	90 (2.2)	56 (0.1)	114 (0.6)
Anterior uveitis	1629 (21.4)	743 (18.5)	254 (0.7)	95 (0.5)
Inflammatory bowel disease	468 (6.2)	271 (6.8)	316 (0.8)	160 (0.8)
Psoriasis	230 (3.0)	148 (3.7)	577 (1.5)	265 (1.3)
Medical treatment				
DMARDs	2444 (32.1)	1233 (30.8)	321 (0.8)	196 (1.0)
TNF inhibitors	1121 (14.7)	527 (13.2)	30 (0.1)	17 (0.1)
sDMARDs	1786 (23.5)	951 (23.7)	310 (0.8)	191 (1.0)
Peroral glucocorticoids	1325 (17.4)	796 (19.9)	1212 (3.2)	891 (4.4)
1 prescription	516 (6.8)	363 (9.1)	687 (1.8)	532 (2.7)
≥2 prescriptions	809 (10.6)	433 (10.8)	525 (1.4)	359 (1.8)
Bisphosphonate*	168 (2.2)	178 (4.4)	118 (0.3)	221 (1.1)
Calcium, vitamin D	472 (6.2)	489 (12.2)	323 (0.8)	606 (3.0)
Oestrogens	0 (0.0)	448 (11.2)	1 (0.0)	1533 (7.7)

The data are presented as n (%) if not stated otherwise. Prior medical conditions were identified in the National Patient Register within 6 years before start of follow-up. Medical treatment with ≥1 prescription identified in the Prescribed Drug Register (irrespective of indication) or the Swedish Rheumatology Quality Register within 1 year before start of follow-up.

*Intravenously administered bisphosphonate not captured in PDR.

AS, ankylosing spondylitis; DMARDs, disease modifying anti-rheumatic drugs; PDR, Prescribed Drug Register; sDMARDs, synthetic DMARDs; TNF, tumour necrosis factor.

### Incidence of non-vertebral fracture

**Figure 1:**
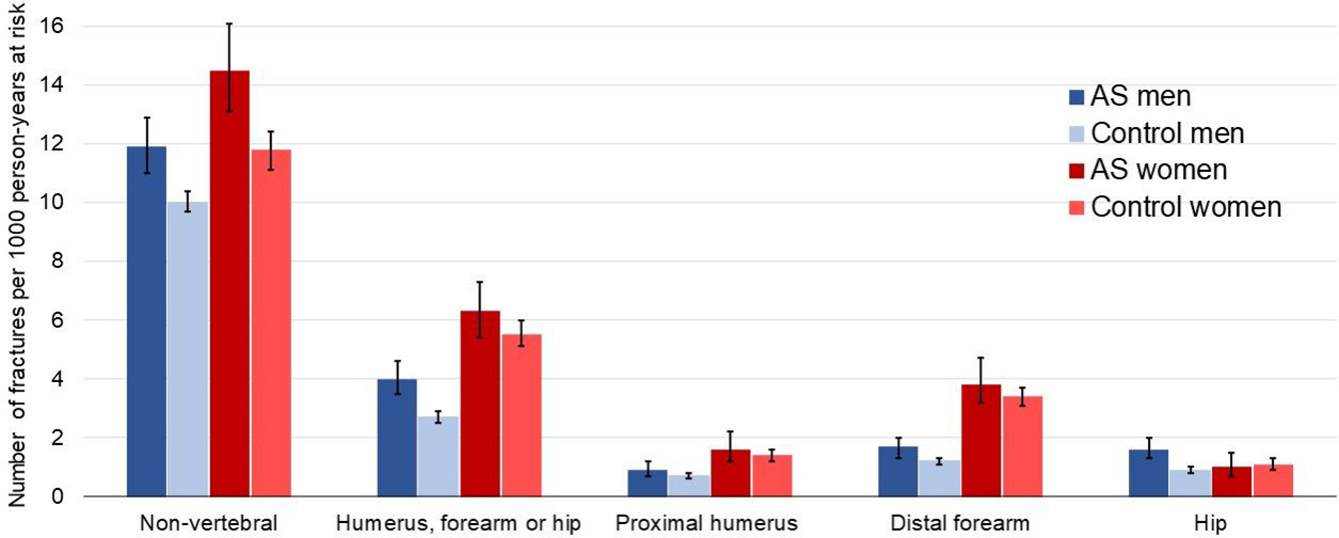
Sex-stratified IRs with 95% CI in AS and matched controls. AS, ankylosing spondylitis; IRs, incidence rates.

**Figure 2:**
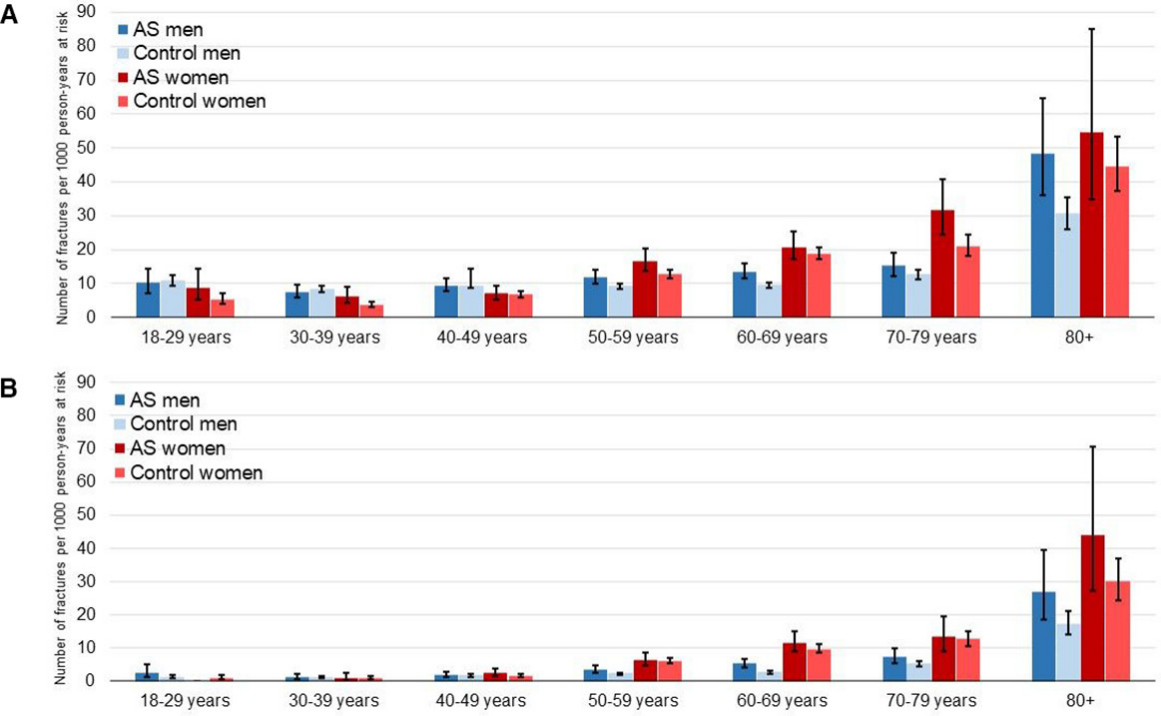
Sex-adjusted and age-stratified IRs for (A) non-vertebral fracture, (B) fracture of the humerus, forearm or hip. AS, ankylosing spondylitis; IRs, incidence rate.

**Figure 3:**
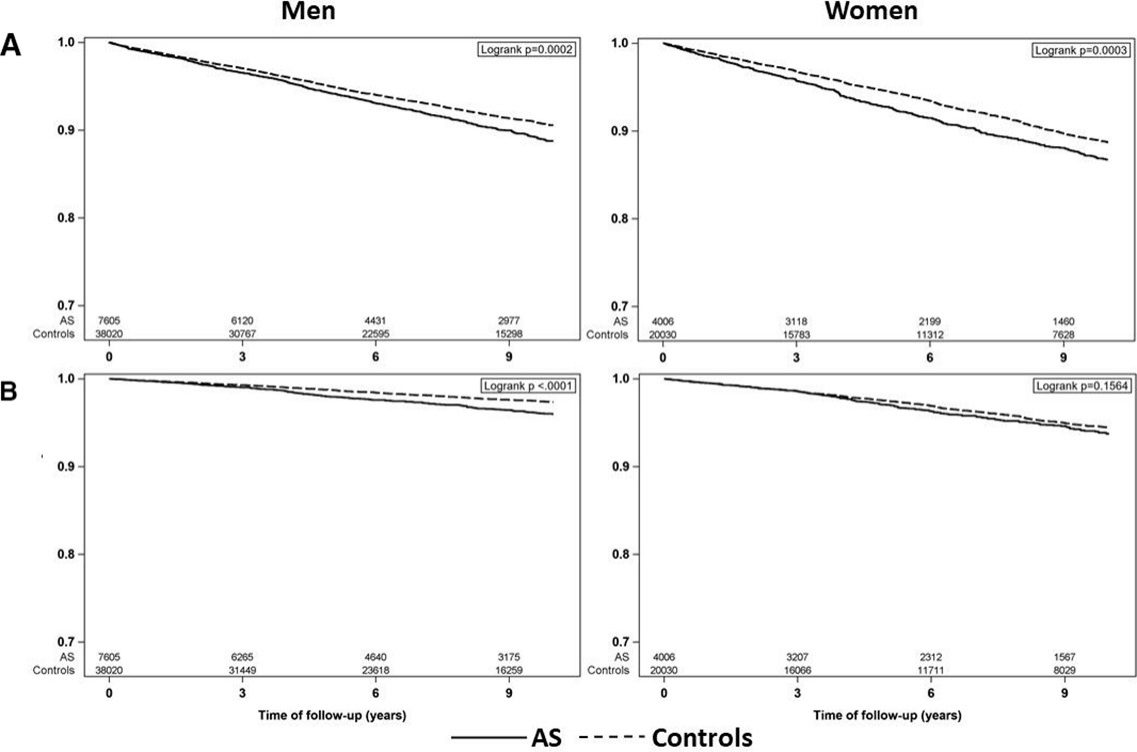
Kaplan-Meier curve for (A) non-vertebral fracture and (B) fracture of the humerus, forearm or hip. AS, ankylosing spondylitis.

**Figure 4:**
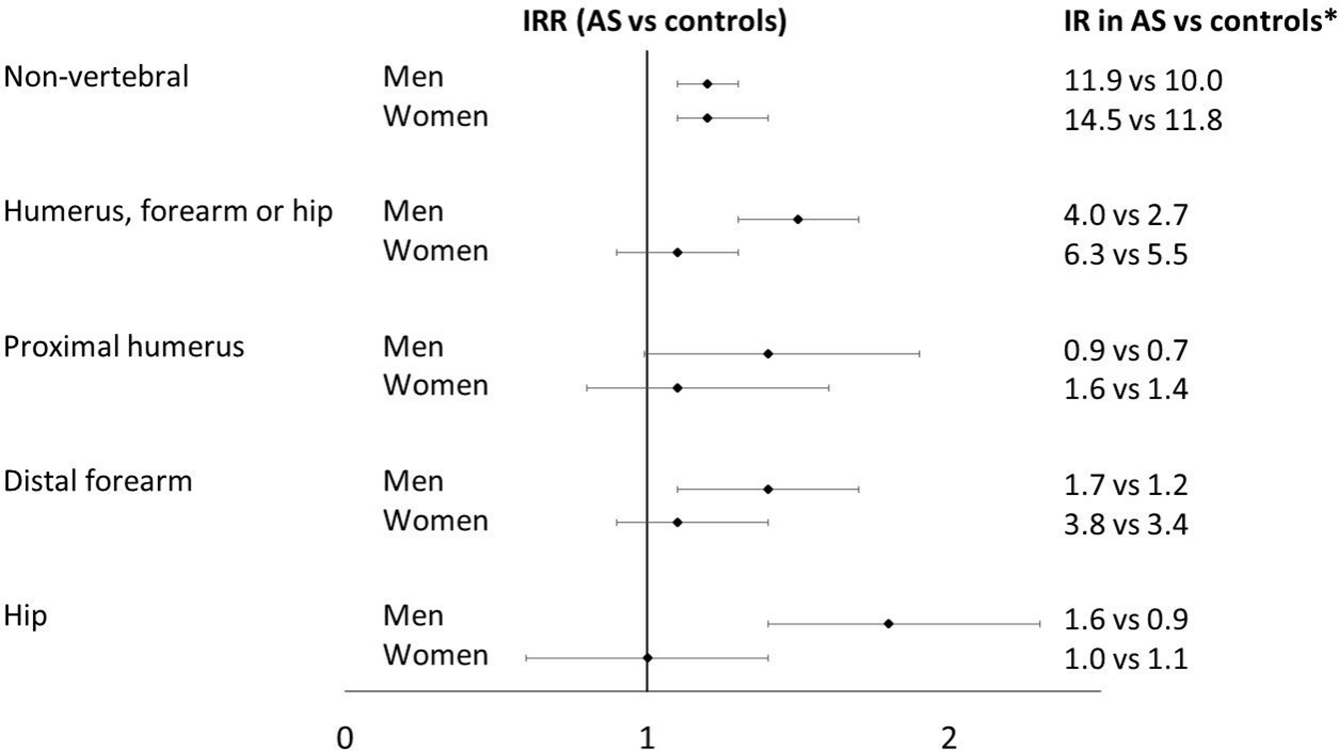
IRRs and corresponding IR point estimate for the studied fracture outcomes in AS versus controls. Sex-stratified IRRs with 95% CI and corresponding incidence rate (IR) point estimates for the five different non-vertebral fracture outcomes in 11 611 patients with AS vs 58 050 age-matched, sex-matched and geography-matched controls (reference) followed prospectively 2007–2016 in the Swedish healthcare and population registers. The IRRs are adjusted for history of prior fracture at study entry. *IRs are presented as number of fractures per 1000 person-years at risk. AS, ankylosing spondylitis; IRRs, IR ratios;

During follow-up, 974 patients with AS and 4106 matched controls experienced an incident non-vertebral fracture. The corresponding IRs with 95% CI were 12.8 (12.0 to 13.6) non-vertebral fractures per 1000 person-years at risk in AS and 10.6 (10.3 to 11.0) in matched controls. The IRs are reported in table 2 and visualised in figure 1 and figure 2A, stratified by sex and age groups. Prior fractures were observed in 140 (14.4%) patients and 486 (11.8%) controls with an incident non-vertebral fracture. The Kaplan-Meier curve showed a lower fracture-free survival in AS patients than in controls (figure 3A). Poisson regression analyses demonstrated slightly higher adjusted IRRs (AS vs controls), both overall (IRR 1.2, 95% CI 1.1 to 1.3) and in sex-stratified models (table 2, figure 4). In men, a significant interaction was found between AS status and age. Accordingly, higher IRRs (AS vs controls) were observed only in men aged 50 years and older, and not in the younger age groups (online supplemental table 2).

10.1136/rmdopen-2022-002753.supp2 Supplementary data

**Table 2:** IRs and IRRs of the studied non-vertebral fracture outcomes in AS and matched controls

	Men		Women	
	AS	Matched controls	AS	Matched controls
Non-vertebral fracture				
Events, n	603	2572	371	1534
IRs with 95% CI	11.9 (11.0 to 12.9)	10.0 (9.7 to 10.4)	14.5 (13.1 to 16.1)	11.8 (11.1 to 12.4)
IRRs with 95% CI, crude	**1.2 (1.1 to 1.3**)	Ref	**1.2 (1.1 to 1.4**)	Ref
IRRs with 95% CI, adjusted*	**1.2 (1.1 to 1.3**)	Ref	**1.2 (1.1 to 1.4**)	Ref
Fracture of the humerus, forearm or hip				
Events, n	210	703	166	741
IRs with 95% CI	4.0 (3.5 to 4.6)	2.7 (2.5 to 2.9)	6.3 (5.4 to 7.3)	5.5 (5.1 to 6.0)
IRRs with 95% CI, crude	**1.5 (1.3 to 1.8**)	Ref	1.1 (0.95 to 1.3)	Ref
IRRs with 95% CI, adjusted*	**1.5 (1.3 to 1.7**)	Ref	1.1 (0.9 to 1.3)	Ref
Proximal humerus fracture				
Events, n	50	182	44	191
IRs with 95% CI	0.9 (0.7 to 1.2)	0.7 (0.6 to 0.8)	1.6 (1.2 to 2.2)	1.4 (1.2 to 1.6)
IRRs with 95% CI, crude	**1.4 (1.0 to 1.9**)	Ref	1.2 (0.8 to 1.6)	Ref
IRRs with 95% CI, adjusted*	1.4 (0.99 to 1.9)	Ref	1.1 (0.8 to 1.6)	Ref
Distal forearm fracture				
Events, n	87	320	103	457
IRs with 95% CI	1.7 (1.3 to 2.0)	1.2 (1.1 to 1.3)	3.8 (3.2 to 4.7)	3.4 (3.1 to 3.7)
IRRs with 95% CI, crude	**1.4 (1.1 to 1.7**)	Ref	1.1 (0.9 to 1.4)	Ref
IRRs with 95% CI, adjusted*	**1.4 (1.1 to 1.7**)	Ref	1.1 (0.9 to 1.4)	Ref
Hip fracture				
Events, n	85	230	28	143
IRs with 95% CI	1.6 (1.3 to 2.0)	0.9 (0.8 to 1.0)	1.0 (0.7 to 1.5)	1.1 (0.9 to 1.3)
IRRs with 95% CI, crude	**1.9 (1.5 to 2.4**)	Ref	1.0 (0.7 to 1.5)	Ref
IRRs with 95% CI, adjusted*	**1.8 (1.4 to 2.3**)	Ref	1.0 (0.6 to 1.4)	Ref

IRs are presented as number of fractures per 1000 person-years at risk with 95% CI. Statistically significant values are written in bold.

*Adjusted for history of prior fracture at study entry.

AS, ankylosing spondylitis; IRRs, IR ratios; IRs, incidence rates; Ref, reference.

### Incidence of fracture of the proximal humerus, distal forearm and hip

During follow-up, 376 patients with AS-matched and 1444-matched controls experienced an incident fracture of the humerus, fracture or hip. The IRs for fracture of the humerus, forearm or hip were 4.8 (95% CI 4.3 to 5.3) fractures per 1000 person-years at risk in AS and 3.6 (95% CI 3.4 to 3.8) in controls, resulting in an increased adjusted IRR (1.3, 95% CI 1.2 to 1.4) in AS versus controls. There was a significant interaction between AS status and sex. In sex-stratified analyses, the IRRs were statistically elevated in the male comparison (IRR 1.5, 95% CI 1.3 to 1.7) but not in the female (IRR 1.1, 95% CI 0.9 to 1.3).

The IRs and IRRs for the combined outcome (fracture of the humerus, forearm or hip) and the separate proximal humerus fracture, distal forearm fracture and hip fracture outcomes are reported in table 2, figures 1 and 4. The highest IRs were noted for forearm fracture in both AS and controls. Women had higher IR point estimates than men for all outcomes except for hip fracture. Overall, the IRRs for fractures of the humerus, forearm and hip were 1.2 (0.99–1.6), 1.2 (1.0–1.4) and 1.5 (1.2–1.8), respectively. The analysis for hip fracture also showed a significant interaction between AS status and sex. In sex-stratified analyses, the IRR for hip fracture was significantly increased only in men with AS in comparison to controls (IRR 1.8, 95% CI 1.4 to 2.3). We found no statistically significant interaction between AS status and age for any of the humerus, forearm and hip outcomes. However, the IRs were low in the younger age groups (figure 2B) and for both hip and humerus fracture, less than 10 AS patients aged 18–49 years experienced a fracture outcome (online supplemental table 2).

### Incidence of secondary fracture outcomes

IRs and IRRs for the first fracture per site based on the two-digit system (S22, S32, S42, S52, S62, S72, S82, S92) are presented in online supplemental table 3. Women with AS (vs matched controls) had statistically significantly increased adjusted IRRs for non-vertebral fractures of the thoracic region (IRR 1.9), pelvis region (IRR 1.8) and shoulder/upper arm region (IRR 1.4). Men with AS (vs matched controls) had statistically significantly increased IRRs for fractures of the forearm region (IRR 1.3) and hip/femur region (IRR 1.8).

10.1136/rmdopen-2022-002753.supp3 Supplementary data

### Sensitivity analyses

We calculated IRs and IRRs in the subset of patients and controls without any prior fracture at baseline. The IRs were in general somewhat lower, but the overall IRR point estimates were unchanged for non-vertebral fractures, fracture of the humerus, forearm or hip, forearm fracture and only marginally changed for humerus fracture (IRR 1.3). For hip fracture, slightly higher IRRs were noted overall (IRR 1.7, 95% CI 1.4 to 2.1), and separately in men (IRR 1.9, 95% CI 1.5 to 2.5) and in women (IRR 1.3, 95% CI 0.8 to 2.0) with AS versus matched controls.

The second sensitivity analysis required 12 months (instead of 5 months as in the main analysis) between fractures at the same site before start of follow-up to be defined as a new incident fracture during follow-up. This change resulted in 23 less non-vertebral fractures and 3 less fractures of the humerus, forearm and hip with only discrete changes (≤0.1 per 1000 person-years at risk) of the overall and sex-stratified IR point estimates from the main analyses and with identical crude IRR point estimates.

Lastly, the vertebral fracture outcome analysis demonstrated elevated adjusted IRR (4.2 (95% CI 3.6 to 4.8)) in AS versus matched controls (online supplemental table 4).

10.1136/rmdopen-2022-002753.supp4 Supplementary data

### Supplemental materials

All used ICD, ATC and procedure codes are presented in online supplemental table 1. Number of fractures, person-years at risk, IRs and IRRs stratified by sex and age groups are presented in online supplemental table 2. A number of fractures, IRs and IRRs for the secondary fracture outcomes are presented in online supplemental table 3 and for the vertebral fracture outcome (sensitivity analysis) in online supplemental table 4.

## Discussion

In this nationwide register-based study, we found a slightly higher risk for non-vertebral fractures in both men (aged 50 years and older) and women with AS compared with controls from the general population. For men with AS, we also demonstrated a statistically significantly higher risk for fracture of the proximal humerus, distal forearm or hip, which was especially pronounced for hip fracture, where the risk was nearly doubled in comparison to male controls.

Previous studies on risk of non-vertebral fractures in AS patients are mainly case–control studies (online supplemental table 5). However, there is one prior Spanish cohort study with a similar design as our present study. Muñoz-Ortego *et al* identified AS patients (n=6474, 66% men, mean age 46 (SD 16)) and matched controls from a primary healthcare database and found a relative risk of non-vertebral fractures similar to our results (HR 1.2 (95% CI 1.0 to 1.4)).^18^ They further adjusted their analyses for body mass index, tobacco smoking, alcohol consumption and peroral glucocorticoids without altering the results, but did not present sex-stratified results nor subcategories of non-vertebral fractures. Three previous case–control studies have used healthcare data registers to identify cases with fracture outcomes and controls without fractures and then assessed if AS was associated with any of the studied fracture outcomes.^16 17 19^ Prieto-Alhambra *et al* found a significant association between AS and non-vertebral fractures in age-matched and sex-matched analyses (OR 1.4 (95% CI 1.1 to 1.7)) but not in multivariable adjusted models including among others fracture history, use of non-steroidal anti-inflammatory drugs and oral corticosteroids.^17^ Vosse *et al* used data from the General Practice Research Database in UK and did not find a statistically significant association between AS and forearm fracture (crude OR 1.3 (95% CI 0.9 to 1.8)) or between AS and hip fracture (crude OR 1.0 (95% CI 0.6 to 1.7)).^16^ However, only 25 patients with AS sustained a hip fracture and the study did not report sex-stratified results. On the contrary, a previous case–control study from Sweden investigating risk of hip or vertebral fracture in different rheumatic disorders found a statistically significant association with AS and hip fracture (OR 2.5 (95% CI 1.9 to 3.1)).^19^ Importantly, they only identified hospitalised patients with AS prior to the hip fracture, which might have influenced the results and generalisability. The studies described above have also presented results for vertebral fractures and, in line with our sensitivity analysis of vertebral fracture, with relative risk estimates considerably higher (HR/OR point estimates ranging between 2.0 and 7.1) than for the non-vertebral fractures.^16–19^ To sum up, the present study is consistent with previous studies except for hip fracture. Furthermore, we give a more detailed description by reporting the risk of non-vertebral fractures by different fracture subtypes, sex and age groups.

10.1136/rmdopen-2022-002753.supp5 Supplementary data

Risk factors in the general population for osteoporotic fractures are among others advancing age, female sex (postmenopausal) and low BMD.^26^ The present study found a higher risk of fractures of the proximal humerus, distal forearm or hip (sites commonly associated with osteoporotic fractures) only in men with AS (vs controls) with an especially increased relative risk for hip fractures. This could imply that the AS disease per se is a more important risk factor for at least hip fractures in men than in women. Importantly, the absolute risks (IRs) were higher in women than men for all studied fracture outcomes except for hip fracture. Also, women with AS (vs control) had a higher risk of non-vertebral fracture and some of the site-specific fractures (online supplemental table 3) and the found sex difference need to be interpreted with caution. Prior cohort studies investigating osteoporosis or low BMD in AS have looked for different AS-related risk factors. Disease duration, Bath AS Disease Activity Index, Bath AS Metrology Index, syndesmophytes, hip involvement and inflammatory parameters have been proposed as risk factors for osteoporosis or low BMD in AS.^10 27–29^ Moreover, markers of inflammation (C reactive protein and erythrocyte sedimentation rate, respectively) were predictors in AS for decrease in femoral neck BMD.^29–31^ Notably, male sex is a risk factor for more severe AS with regard to structural AS-related skeletal alterations.^32 33^

Fracture is a possible consequence of a fall. The slightly increased risk of any non-vertebral fracture could partly be explained by an increased fall tendency in AS patients in comparison to general population. A recently published review of falls in patients with SpA reported a prevalence of falls during a retrospective 12-month period of 13%–25%.^34^ Risk factors for falls in SpA was among others functional limitation, measured by Bath AS Functional Index (BASFI), and reduced spinal mobility, measured by BASMI. A prior study of 40 patients with AS and 40 age-matched and sex-matched controls found a statistically significantly higher proportion with a history of falls in AS (20%) compared with the controls (5%).^35^

### Limitations

The study has limitations, mainly due to the register-based design. First, we cannot exclude misclassification of AS and the fracture outcomes. However, a previous validation study of diagnostic codes for AS in NPR has demonstrated reasonably high positive predictive values, 70% and 89%, respectively, for fulfilling the modified New York criteria or any set of SpA criteria.^36^ Also, the AS cohort included in the present study had an expected proportion of anterior uveitis and IBD, but a somewhat lower proportion of psoriasis, the latter probably explained by the exclusion of patients with a parallel PsA.^37^ In a single-centre validation study of diagnostic codes in NPR for humerus fractures (ICD 10: S422-S424), 5% of the visits were erroneously coded and did not represent a humerus fracture.^38^ Another study has compared data from the Swedish hip fracture register (SHR), and NPR.^39^ In total 98% of the hip fractures registered in SHR were also found in NPR but only 70% had a combination with an appropriate surgical procedure code. This could implicate an underestimation of the IRs in the present study, but would not have such effect on the IRRs. Second, both fractures caused by low and high energy trauma were included in the fracture outcomes. Third, we could not identify the broader concept of axial SpA since non-radiographic axial SpA cannot be distinguished from peripheral SpA by ICD-10 codes. Fourth, patients with AS without any visits to the specialised rheumatology or internal medicine outpatient care during the study period, presumably less severe cases, were not captured. Fifth, low number of fractures of the humerus, forearm and hip hampered the statistical power in the younger age groups. Similarly, we cannot rule out a type 2 error in the female comparison of fractures of the humerus, forearm and hip since a small difference is harder to detect due to the combination of less women than men included in the study and generally higher IRs for the outcomes in women. Sixth, potential time trends in fracture risk during the follow-up period 2007–2016 were not specifically addressed. Seventh, observation studies such as ours can be influenced by surveillance biases, which would be the case if patients with AS were more (or less) thoroughly investigated for the studied outcome than the controls. However, contrary to vertebral fractures, non-vertebral fractures in most cases come into medical attention. Lastly, the influence of possible confounders and other disease associated factors were beyond the objective of the present study and was not investigated. With the present study design, an attempt to adjust for explanatory factors would be complicated by lack of important data such as AS severity and disease activity likewise smoking habits, and the risk of confounding by indication for treatment exposure. There is also a potential risk of adjusting away a true association with the AS disease per se.

## Conclusions

Both men and women with AS have a slightly higher risk of non-vertebral fractures than the general population. A statistically significantly higher risk of fractures of the proximal humerus, distal forearm or hip was found in men with AS in comparison to general population, where the relative risk was especially pronounced for hip fracture.

## Data Availability

All data relevant to the study are included in the article or uploaded as online supplemental information. The data sets generated and/or analyzed during the present study are not publicly available due to the General Data Protection Regulation.
