# Supplementary material for: Occurrence and relative risks for non-vertebral fractures in patients with ankylosing spondylitis compared with the general population: a register-based study from Sweden
Source: RMD Open. 2023 Feb 14;9(1):e002753. doi: 10.1136/rmdopen-2022-002753 (PMC9930563; doi:10.1136/rmdopen-2022-002753)
Supplement: Supplementary data [file rmdopen-2022-002753supp001.pdf]

Supplemental Table 1. ICD and ATC codes used to identify the patients, fracture outcomes, medical conditions, and treatments.

National Patient Register (NPR)	ICD-10	Surgical procedure codes
**Case definition**		
Ankylosing spondylitis	M45	
**Fracture outcomes**		
Non-vertebral fracture (except skull, finger, toe)	S222, S223, S224, S225, S321, S322, S323, S324, S325, S42, S52, S620, S621, S622, S623, S624, S72, S82, S920, S921, S922, S923, S927	
Fracture of humerus, forearm or hip	S422, S525, S526, S720*, S721*, S722*	NFB, NFJ39-NFJ99
Proximal humerus fracture	S422	
Distal forearm fracture	S525, S526	
Hip fracture	S720*, S721*, S722*	NFB, NFJ39-NFJ99
Vertebral fracture (sensitivity analysis)	M485, S120, S121, S122, S220, S221, S320	
**Other medical conditions**		
Any fracture (except skull, finger, toe)	S12, S22, S32, S42, S52, S620, S621, S622, S623, S624, S628, S72, S82, S920, S921, S922, S923, S927, S929, T02, T08, T10, T12, T142, M485, M800A, M800J, M800K	
Osteoporosis	M80, M81, M82	
Fall injury	S00-T14 + W00-W19 (type of trauma indicating a fall)	
Anterior uveitis	H20, H221	
Inflammatory bowel disease	K50, K51	
Psoriasis	L40 and/or an ATC-code for anti-psoriatic drugs	
**Prescribed Drug Register (PDR)**	**ATC**	
**AS related**		
DMARDs	L04AB01, L04AB02, L04AB04, L04AB05, L04AB06, L04AX01, L04AA13, L04AD01, M01CB01, M01CB03, P01BA01, P01BA02, L04AA32, L01BA01, L04AX03, A07EC01	
TNF inhibitors	L04AB01, L04AB02, L04AB04, L04AB05, L04AB06	
sDMARDs	L04AX01, L04AA13, L04AD01, M01CB01, M01CB03, P01BA01, P01BA02, L04AA32, L01BA01, L04AX03, A07EC01	
Peroral glucocorticoids	H02AB (only peroral administration)	
Anti-psoriatic drugs	D05AX, D05B	
**Osteoporosis related**		
Active osteoporosis treatment	G03XC, H05AA02, M05BA, M05BB, M05BX03, M05BX04	
Bisphosphonate	M05BA, M05BB	
Calcium, D vitamin	A12AX or A11CC + A12AA	
Estrogens	G03C	

*Codes S720, S721 and S722 require a combination with any of the specified surgical procedure codes to represent a hip fracture.

DMARDs, disease modifying anti-rheumatic drugs; SD, standard deviation; sDMARDs, synthetic DMARDs; TNF tumor necrosis factor
