# Supplementary material for: Occurrence and relative risks for non-vertebral fractures in patients with ankylosing spondylitis compared with the general population: a register-based study from Sweden
Source: RMD Open. 2023 Feb 14;9(1):e002753. doi: 10.1136/rmdopen-2022-002753 (PMC9930563; doi:10.1136/rmdopen-2022-002753)
Supplement: Supplementary data [file rmdopen-2022-002753supp002.pdf]

Supplemental Table 2. IR and IRRs, stratified by sex and age groups for the different non-vertebral fracture outcomes.

	AS			Matched controls			
	Event	PYs	IR	Event	PYs	IR	**IRR, adjusted**
**Non-vertebral fracture**	974	76 270	12.8 (12.0–13.6)	4106	386 499	10.6 (10.3–11.0)	1.2 (1.1–1.3)
Men	603	50 668	11.9 (11.0–12.9)	2572	256 076	10.0 (9.7–10.4)	1.2 (1.1–1.3) *
- 18 to 29 years	31	3052	10.2 (7.1–14.4)	161	15 020	10.7 (9.3–12.4)	1.0 (0.6–1.4)
- 30 to 39 years	58	7747	7.5 (5.8–9.7)	316	38 340	8.2 (7.4–9.2)	0.9 (0.7–1.2)
- 40 to 49 years	106	11 187	9.5 (7.8–11.5)	521	55 742	9.3 (8.6–10.2)	1.0 (0.8–1.3)
- 50 to 59 years	140	11 811	11.9 (10.1–14.0)	548	59 210	9.3 (8.5–10.1)	1.3 (1.1–1.5)
- 60 to 69 years	150	11 167	13.4 (11.5–15.8)	550	57 584	9.6 (8.8–10.4)	1.3 (1.1–1.6)
- 70 to 79 years	73	4772	15.3 (12.2–19.2)	314	24 863	12.6 (11.3–14.1)	1.1 (0.9–1.5)
- 80+	45	931	48.3 (36.1–64.6)	162	5317	30.5 (26.1–35.6)	1.6 (1.1–2.2)
Women	371	25 602	14.5 (13.1–16.1)	1534	130 423	11.8 (11.1–12.4)	1.2 (1.1–1.4)
- 18 to 29 years	15	1716	8.7 (5.3–14.5)	45	8586	5.2 (3.9–7.0)	1.6 (0.9–2.9)
- 30 to 39 years	26	4175	6.2 (4.2–9.1)	78	20 882	3.7 (3.0–4.7)	1.7 (1.1–2.6)
- 40 to 49 years	46	6481	7.1 (5.3–9.5)	218	32 556	6.7 (5.9–7.7)	1.1 (0.8–1.4)
- 50 to 59 years	102	6090	16.7 (13.8–20.3)	398	30 922	12.9 (11.7–14.2)	1.3 (1.0–1.6)
- 60 to 69 years	101	4858	20.8 (17.1–25.3)	475	25 129	18.9 (17.3–20.7)	1.1 (0.9–1.3)
- 70 to 79 years	60	1895	31.7 (24.5–40.9)	208	9838	21.1 (18.3–24.5)	1.5 (1.1–2.0)
- 80+	21	386	54.5 (34.9–85.1)	112	2510	44.6 (37.4–53.3)	1.2 (0.7–1.9)
**Fracture of humerus, forearm or hip**	376	78 828	4.8 (4.3–5.3)	1444	397 455	3.6 (3.4–3.8)	1.3 (1.2–1.4) #
Men	210	52 316	4.0 (3.5–4.6)	703	263 908	2.7 (2.5–2.9)	1.5 (1.3–1.7)
- 18 to 29 years	8	3098	2.6 (1.3–5.2)	19	15 415	1.2 (0.8–1.9)	-
- 30 to 39 years	10	7907	1.3 (0.7–2.4)	48	39 320	1.2 (0.9–1.6)	1.0 (0.5–2.1)
- 40 to 49 years	22	11 542	1.9 (1.3–2.9)	98	57 479	1.7 (1.4–2.1)	1.1 (0.7–1.8)
- 50 to 59 years	44	12 233	3.6 (2.7–4.8)	142	61 119	2.3 (2.0–2.7)	1.5 (1.1–2.1)
- 60 to 69 years	63	11 627	5.4 (4.2–6.9)	165	59 348	2.8 (2.4–3.2)	1.9 (1.4–2.5)
- 70 to 79 years	36	4909	7.3 (5.3–10.1)	135	25 677	5.3 (4.5–6.2)	1.3 (0.9–1.9)
- 80+	27	1000	27.0 (18.5–39.5)	96	5549	17.3 (14.1–21.3)	1.5 (0.98–2.4)
Women	166	26 512	6.3 (5.4–7.3)	741	133 547	5.5 (5.1–6.0)	1.1 (0.9–1.3)
- 18 to 29 years	0	1750	0.0	9	8688	1.0 (0.5–2.0)	-
- 30 to 39 years	4	4247	0.9 (0.4–2.5)	20	21 097	0.9 (0.6–1.5)	-
- 40 to 49 years	16	6622	2.4 (1.5–3.9)	54	33 048	1.6 (1.2–2.1)	1.5 (0.8–2.6)
- 50 to 59 years	41	6340	6.5 (4.8–8.8)	193	31 765	6.1 (5.3–7.0)	1.1 (0.8–1.5)
- 60 to 69 years	60	5121	11.7 (9.1–15.1)	255	26 058	9.8 (8.6–11.1)	1.2 (0.9–1.6)
- 70 to 79 years	27	2024	13.3 (9.1–19.5)	130	10 237	12.7 (10.7–15.0)	1.0 (0.7–1.6)
- 80+	18	409	44.0 (27.4–70.7)	80	2654	30.1 (24.5–37.1)	1.4 (0.8–2.5)
**Proximal humerus fracture**	94	79 857	1.2 (1.0–1.4)	373	401 627	0.9 (0.8–1.0)	1.2 (0.99–1.6)
Men	50	52 871	0.9 (0.7–1.2)	182	265 825	0.7 (0.6–0.8)	1.4 (0.99–1.9)
- 18 to 29 years	2	3116	0.6 (0.2–2.6)	1	15 459	0.1 (0.0–0.5)	-
- 30 to 39 years	2	7940	0.3 (0.1–1.0)	5	39 489	0.1 (0.1–0.3)	-
- 40 to 49 years	4	11 590	0.3 (0.1–0.9)	27	57 766	0.5 (0.3–0.7)	-
- 50 to 59 years	10	12 354	0.8 (0.4–1.5)	48	61 473	0.8 (0.6–1.0)	1.0 (0.5–2.0)
- 60 to 69 years	19	11 795	1.6 (1.0–2.5)	47	59 870	0.8 (0.6–1.0)	1.9 (1.1–3.3)
- 70 to 79 years	5	4988	1.0 (0.4–2.4)	36	26 047	1.4 (1.0–1.9)	-
- 80+	8	1087	7.4 (3.7–14.7)	18	5720	3.1 (1.9–5.1)	-
Women	44	26 986	1.6 (1.2–2.2)	191	135 801	1.4 (1.2–1.6)	1.1 (0.8–1.6)
- 18 to 29 years	0	1750	0.0	0	8719	0.0	-
- 30 to 39 years	1	4252	0.2 (0.0–1.7)	3	21 153	0.1 (0.0–0.4)	-
- 40 to 49 years	3	6665	0.5 (0.1–1.4)	10	33 210	0.3 (0.2–0.6)	-

- 50 to 59 years	7	6444	1.1 (0.5–2.3)	43	32 265	1.3 (1.0–1.8)	-
- 60 to 69 years	21	5270	4.0 (2.6–6.1)	72	26 894	2.7 (2.1–3.4)	1.5 (0.9–2.4)
- 70 to 79 years	8	2128	3.8 (1.9–7.6)	40	10 710	3.7 (2.8–5.1)	-
- 80+	4	478	8.4 (3.1–22.3)	23	2851	8.1 (5.4–12.0)	-
**Distal forearm fracture**	190	79 460	2.4 (2.1–2.8)	777	399 604	1.9 (1.8–2.1)	1.2 (1.0–1.4)
Men	87	52 676	1.7 (1.3–2.0)	320	265 087	1.2 (1.1–1.3)	1.4 (1.1–1.7)
- 18 to 29 years	6	3101	1.9 (0.9–4.3)	18	15 416	1.2 (0.7–1.8)	-
- 30 to 39 years	6	7926	0.8 (0.3–1.7)	39	39 350	1.0 (0.7–1.4)	-
- 40 to 49 years	19	11 553	1.6 (1.0–2.6)	65	57 587	1.1 (0.9–1.4)	1.5 (0.9–2.5)
- 50 to 59 years	21	12 304	1.7 (1.1–2.6)	78	61 312	1.3 (1.0–1.6)	1.3 (0.8–2.1)
- 60 to 69 years	25	11 749	2.1 (1.4–3.1)	67	59 703	1.1 (0.9–1.4)	1.9 (1.2–2.9)
- 70 to 79 years	5	4973	1.0 (0.4–2.4)	38	25 996	1.5 (1.1–2.0)	-
- 80+	5	1071	4.7 (1.9–11.2)	15	5724	2.6 (1.6–4.3)	-
Women	103	26 784	3.8 (3.2–4.7)	457	134 517	3.4 (3.1–3.7)	1.1 (0.9–1.4)
- 18 to 29 years	0	1750	0.0	9	8688	1.0 (0.5-2.0)	-
- 30 to 39 years	3	4248	0.7 (0.2–2.2)	16	21 105	0.8 (0.5–1.2)	-
- 40 to 49 years	13	6639	2.0 (1.1–3.4)	44	33 068	1.3 (1.0–1.8)	1.5 (0.8–2.7)
- 50 to 59 years	33	6362	5.2 (3.7–7.3)	140	31 923	4.4 (3.7–5.2)	1.2 (0.8–1.7)
- 60 to 69 years	33	5226	6.3 (4.5–8.9)	168	26 391	6.4 (5.5–7.4)	1.0 (0.7–1.4)
- 70 to 79 years	12	2103	5.7 (3.2–10.1)	59	10 509	5.6 (4.3–7.3)	1.0 (0.5–1.8)
- 80+	9	456	19.7 (10.2–38.2)	21	2834	7.4 (4.9–11.2)	-
**Hip fracture**	113	79 848	1.4 (1.2–1.7)	373	401 934	0.9 (0.8–1.0)	1.5 (1.2–1.8) #
Men	85	52 791	1.6 (1.3–2.0)	230	265 859	0.9 (0.8–1.0)	1.8 (1.4–2.3)
- 18 to 29 years	0	3119	0.0	0	15 460	0.0	-
- 30 to 39 years	2	7952	0.3 (0.1–1.0)	4	39 491	0.1 (0.0–0.3)	-
- 40 to 49 years	0	11 603	0.0	6	57 836	0.1 (0.0-0.2)	-
- 50 to 59 years	13	12 357	1.1 (0.6–1.8)	20	61 572	0.3 (0.2–0.5)	3.1 (1.5–6.2)
- 60 to 69 years	22	11 766	1.9 (1.2–2.8)	61	59 885	1.0 (0.8–1.3)	1.7 (1.0–2.8)
- 70 to 79 years	28	4962	5.6 (3.9–8.2)	69	25 968	2.7 (2.1–3.3)	2.0 (1.3–3.1)
- 80+	20	1032	19.4 (12.5–30.2)	70	5646	12.4 (9.6–16.0)	1.5 (0.9–2.5)
Women	28	27 057	1.0 (0.7–1.5)	143	136 074	1.1 (0.9–1.3)	1.0 (0.6–1.4)
- 18 to 29 years	0	1750	0.0	0	8719	0.0	-
- 30 to 39 years	0	4253	0.0	1	21 159	0.0 (0.0–0.3)	-
- 40 to 49 years	1	6673	0.1 (0.0–1.1)	0	33 231	0.0	-
- 50 to 59 years	1	6457	0.2 (0.0–1.1)	13	32 362	0.4 (0.2-0.7)	-
- 60 to 69 years	7	5349	1.3 (0.6–2.7)	36	27 043	1.3 (1.0-1.8)	-
- 70 to 79 years	10	2123	4.7 (2.5–8.7)	42	10 756	3.9 (2.9–5.3)	1.2 (0.6–2.3)
- 80+	9	452	19.9 (10.2–38.8)	51	2804	18.2 (13.7-24.1)	-

IRs are presented as number of fractures per 1000 person-years at risk. IRRs are adjusted for history of any prior fracture*Significant interaction between AS status and age. # Significant interaction between AS status and sex.

AS, ankylosing spondylitis; PYs, person-years; IRs, incidence rates; IRRs, incidence rate ratios
