# Supplementary material for: Occurrence and relative risks for non-vertebral fractures in patients with ankylosing spondylitis compared with the general population: a register-based study from Sweden
Source: RMD Open. 2023 Feb 14;9(1):e002753. doi: 10.1136/rmdopen-2022-002753 (PMC9930563; doi:10.1136/rmdopen-2022-002753)
Supplement: Supplementary data [file rmdopen-2022-002753supp003.pdf]

Supplemental Table 3. IRs and IRRs of secondary non-vertebral fracture outcomes in AS and matched controls.

	Men		Women	
	AS	Matched controls	AS	Matched controls
**S22 fracture (thoracic region)**				
Events, n	110	493	37	97
IRs with 95% CI	2.1 (1.7-2.5)	1.9 (1.7-2.0)	1.4 (1.0-1.9)	0.7 (0.6-0.9)
IRRs with 95% CI, crude	1.1 (0.9-1.4)§	Ref	**1.9 (1.3-2.8)**	Ref
IRRs with 95% CI, adjusted[#]	1.1 (0.9-1.4)§	Ref	**1.9 (1.3-2.7)**	Ref
**S32 fracture (pelvis region)**				
Events, n	20	86	21	55
IRs with 95% CI	0.4 (0.2-0.6)	0.3 (0.3-0.4)	0.8 (0.5-1.2)	0.4 (0.3-0.5)
IRRs with 95% CI, crude	1.2 (0.7-1.9)	Ref	**1.9 (1.2-3.1)**	Ref
IRRs with 95% CI, adjusted[#]	1.1 (0.7-1.8)	Ref	**1.8 (1.1-3.0)**	Ref
**S42 fracture (shoulder/upper arm)**				
Events, n	113	460	71	259
IRs with 95% CI	2.1 (1.8-2.6)	1.7 (1.6-1.9)	2.6 (2.1-3.3)	1.9 (1.7-2.2)
IRRs with 95% CI, crude	**1.2 (1.0-1.5)**	Ref	**1.4 (1.1-1.8)**	Ref
IRRs with 95% CI, adjusted[#]	1.2 (0.99-1.5)	Ref	**1.4 (1.0-1.8)**	Ref
**S52 fracture (forearm)**				
Events, n	117	446	121	552
IRs with 95% CI	2.2 (1.9-2.7)	1.7 (1.5-1.9)	4.5 (3.8-5.4)	4.1 (3.8-4.5)
IRRs with 95% CI, crude	**1.3 (1.1-1.6)**	Ref	1.1 (0.9-1.3)	Ref
IRRs with 95% CI, adjusted[#]	**1.3 (1.1-1.6)**	Ref	1.1 (0.9-1.3)	Ref
**S62 fracture (hand except finger)**				
Events, n	72	384	35	121
IRs with 95% CI	1.4 (1.1-1.7)	1.4 (1.3-1.6)	1.3 (0.9-1.8)	0.9 (0.7-1.1)
IRRs with 95% CI, crude	0.9 (0.7-1.2)	Ref	1.5 (0.99-2.1)	Ref
IRRs with 95% CI, adjusted[#]	0.9 (0.7-1.2)	Ref	1.4 (0.99-2.1)	Ref
**S72 fracture (hip/femur)**				
Events, n	113	313	45	175
IRs with 95% CI	2.1 (1.8-2.6)	1.2 (1.0-1.3)	1.7 (1.2-2.2)	1.3 (1.1-1.5)
IRRs with 95% CI, crude	**1.8 (1.5-2.2)**	Ref	1.3 (0.9-1.8)	Ref
IRRs with 95% CI, adjusted[#]	**1.8 (1.4-2.2)**	Ref	1.2 (0.9-1.7)	Ref
**S82 fracture (lower leg)**				
Events, n	122	531	82	358
IRs with 95% CI	2.3 (1.9-2.8)	2.0 (1.8-2.2)	3.1 (2.5-3.8)	2.7 (2.4-2.9)
IRRs with 95% CI, crude	1.2 (0.95-1.4)	Ref	1.2 (0.9-1.5)	Ref
IRRs with 95% CI, adjusted[#]	1.1 (0.9-1.4)	Ref	1.1 (0.9-1.4)	Ref
**S92 fracture (foot except toe)**				
Events, n	47	242	35	135
IRs with 95% CI	0.9 (0.7-1.2)	0.9 (0.8-1.0)	1.3 (0.9-1.8)	1.0 (0.8-1.2)
IRRs with 95% CI, crude	1.0 (0.7-1.3)	Ref	1.3 (0.9-1.9)	Ref
IRRs with 95% CI, adjusted[#]	1.0 (0.7-1.3)	Ref	1.3 (0.9-1.9)	Ref

IRs are presented as number of fractures per 1000 person-years at risk with 95% confidence interval. Each fracture per site based on the ICD 10 two-digit system (S22, S32, S42, S52, S62, S72, S82, S92) is analyzed separately.

[#] Adjusted for history of prior fracture at study entry. § Interaction between AS and age.

AS, ankylosing spondylitis; CI, confidence interval; IRs, incidence rates; IRRs, incidence rate ratios; Ref, reference.
