# Supplementary material for: Occurrence and relative risks for non-vertebral fractures in patients with ankylosing spondylitis compared with the general population: a register-based study from Sweden
Source: RMD Open. 2023 Feb 14;9(1):e002753. doi: 10.1136/rmdopen-2022-002753 (PMC9930563; doi:10.1136/rmdopen-2022-002753)
Supplement: Supplementary data [file rmdopen-2022-002753supp004.pdf]

**Supplemental Table 4.** IRs and IRRs of the vertebral fracture outcome in AS and matched controls.

| | **AS** | | | **Matched controls** | | | |
|---|---|---|---|---|---|---|---|
| | Event | PYs | IR | Event | PYs | IR | **IRR, adjusted** |
| **Vertebral fracture** | 357 | 79000 | 4.5 (4.1-5.0) | 425 | 401559 | 1.1 (1.0-1.2) | 4.2 (3.6-4.8) |
| Men | 259 | 52139 | 5.0 (4.4-5.6) | 291 | 265537 | 1.1 (1.0-1.2) | 4.5 (3.8-5.3) |
| Women | 98 | 26861 | 3.6 (3.0-4.4) | 134 | 136022 | 1.0 (0.8-1.2) | 3.6 (2.8-4.6) |
| **Vertebral fracture, sensitivity analyses*** | 287 | 74181 | 3.9 (3.4-4.3) | 331 | 355931 | 0.9 (0.8-1.0) | 4.2 (3.6-4.9) |
| Men | 213 | 48895 | 4.4 (3.8-5.0) | 232 | 234446 | 1.0 (0.9-1.1) | 4.4 (3.7-5.3) |
| Women | 74 | 25286 | 2.9 (2.3-3.7) | 99 | 121486 | 0.8 (0.7-1.0) | 3.6 (2.7-4.8) |

IRs are presented as number of fractures per 1000 person-years at risk. IRRs are adjusted for history of any prior fracture.

*Data for the subgroup of patients and their matched controls without a history of any fracture within the six preceding years before start of follow-up.

AS, ankylosing spondylitis; PYs, person-years; IRs, incidence rates; IRRs, incidence rate ratios
