# Supplementary material for: Occurrence and relative risks for non-vertebral fractures in patients with ankylosing spondylitis compared with the general population: a register-based study from Sweden
Source: RMD Open. 2023 Feb 14;9(1):e002753. doi: 10.1136/rmdopen-2022-002753 (PMC9930563; doi:10.1136/rmdopen-2022-002753)
Supplement: Supplementary data [file rmdopen-2022-002753supp005.pdf]

Supplemental Table 5. Previous studies of non-vertebral fractures in AS in comparison to general population.

	Country	Study period	Study design	Outcome	Number of study subjects	Number of fractures	IR	Relative effect size	Relative effect size, adjusted
Cooper et al. [21]	USA	1935-1989 (inclusion)	Cohort	Any limb	121 men with AS<br>37 women with AS	17<br>7	NA	0.9 (0.5-1.4)[1]<br>1.3 (0.5-2.6)[1]	
Munoz-Ortego et al.[18]	Spain	2006-2011	Cohort	Non-vertebral fracture	6474 patients with AS / 32346 controls	218 / 861	8.3 / 6.8	1.21 (1.04-1.41)[2]	1.19 (1.02-1.39) *
Prieto-Alhambra et al.[17]	Denmark	2000	Case-control	Non-vertebral fracture	121291 patients with fracture	121 with AS	NA	1.39 (1.12-1.73)[3]	1.05 (0.84-1.32) #
Weiss et al. [19]	Sweden	1987-2004	Case-control	Hip fracture	47282 with hip fracture	64 with hospitalized AS	NA	2.5 (1.9-3.1)[3]	
Vosse et al. [16]	United Kingdom	1988-1999	Case-control	Forearm fracture<br><br>Hip fracture	44220 with forearm fracture<br>14387 with hip fracture	82 with AS<br><br>25 with AS	NA<br><br>NA	1.30 (0.94-?)[3]<br><br>0.96 (0.56-1.67)[3]	1.21 (0.87-1.69) §<br><br>0.77 (0.43-1.37) §

IRs are presented as number of fractures per 1000 person-years at risk.
[1] Standardized morbidity ratio, [2] Hazard ratio, [3] Odds ratio
*Adjusted for body mass index, tobacco smoking, alcohol consumption and oral corticosteroids.
#Adjusted for fracture history, annual income, social status, working status, educational status, number of consultations to general practitioners and practicing specialists, alcoholism and use of oral NSAIDs and oral corticosteroids.
§Adjusted for a wide range of clinical variables and medication associated with the risk of fracture. Smoking history and body mass index were included if entered in the database.
AS, ankylosing spondylitis; IR, incidence rate; NA, not applicable

1. Cooper C, Carbone L, Michet CJ, Atkinson EJ, O'Fallon WM, Melton LJ, 3rd. Fracture risk in patients with ankylosing spondylitis: a population based study. *The Journal of rheumatology*. 1994;21(10):1877-82.
2. Munoz-Ortego J, Vestergaard P, Rubio JB, Wordsworth P, Judge A, Javaid MK, et al. Ankylosing spondylitis is associated with an increased risk of vertebral and nonvertebral clinical fractures: a population-based cohort study. *J Bone Miner Res*. 2014;29(8):1770-6.
3. Prieto-Alhambra D, Munoz-Ortego J, De Vries F, Vosse D, Arden NK, Bowness P, et al. Ankylosing spondylitis confers substantially increased risk of clinical spine fractures: a nationwide case-control study. *Osteoporos Int*. 2015;26(1):85-91.
4. Weiss RJ, Wick MC, Ackermann PW, Montgomery SM. Increased fracture risk in patients with rheumatic disorders and other inflammatory diseases -- a case-control study with 53,108 patients with fracture. *The Journal of rheumatology*. 2010;37(11):2247-50.
5. Vosse D, Landewe R, van der Heijde D, van der Linden S, van Staa TP, Geusens P. Ankylosing spondylitis and the risk of fracture: results from a large primary care-based nested case-control study. *Annals of the rheumatic diseases*. 2009;68(12):1839-42.
